# Electric-based dressing for wound management

**DOI:** 10.3389/fcimb.2026.1841828

**Published:** 2026-05-08

**Authors:** Yanling Hu, Xiaole Liu, Bin Huang, Hongjin Chen, Yingnan Song, Dongliang Yang

**Affiliations:** 1 College of Life and Health, Nanjing Polytechnic Institute, Nanjing, China; 2 Key Laboratory of Flexible Electronics (KLOFE) and Institute of Advanced Materials (IAM), School of Physical and Mathematical Sciences, Nanjing Tech University (NanjingTech), Nanjing, China; 3 Academy of Integrative Medicine, Fujian Key Laboratory of Integrative Medicine on Geriatrics, Fujian University of Traditional Chinese Medicine, Fuzhou, Fujian, China; 4 Center for Tissue Engineering and Stem Cell Research, Translation Medicine Research Center, Guizhou Biomanufacturing Laboratory, Guizhou Medical University, Guiyang, China

**Keywords:** electret, electrical stimulation, hypertrophic scar, infection, wound healing

## Abstract

Electrical stimulation directs cell behavior via spatially controlled electric fields to enhance tissue regeneration and wound healing. Electrets, a class of functional materials with permanent polarization, offer distinct advantages, such as low cost, stable and sustained electric field output, and excellent biocompatibility, positioning them as promising candidates for therapeutic applications. This minireview summarizes the main recent progress in organic electret-based materials for wound management, including an introduction to wound treatment and advances in electret-based therapy for accelerated wound healing, infection control, and wound monitoring. Finally, the key challenges and future directions for clinical translation and theoretical development are discussed.

## 1 Introduction

The high incidence and severe consequences of skin wounds place a substantial burden on the healthcare system, with chronic non-healing wounds being particularly challenging. (Liu et al., 2025b) As the global population continues to age, the prevalence of diabetes and obesity is on the rise.(Liu et al., 2025b) Combined with the ongoing threat of infectious diseases, wound healing, especially the management of chronic wounds, remains a heavy burden that extends from patients and caregivers to the entire healthcare system. (Uberoi et al., 2024) Hence, there is an urgent need to develop more cost-effective treatment strategies and explore innovative approaches that can accelerate the wound healing process.

To date, a range of wound healing interventions has been developed, including cell therapy, hyperbaric oxygen therapy, negative pressure wound therapy, and localized therapeutics delivery.(Monami et al., 2025; Xue et al., 2025a) While these approaches demonstrate considerable promise, their clinical application is often hindered by high costs, variable efficacy, and biosafety concerns. (Zhang et al., 2023a) It is well established that wound dressings constitute a fundamental and irreplaceable element of clinical care. (Zhang et al., 2025) However, traditional dressings, such as cotton, bandages, and gauze, function primarily as passive physical barriers that shield wounds from contamination, lacking the capacity to actively participate in the healing process. Moreover, the matrix materials of these conventional dressings typically exhibit no intrinsic biological activity (e.g., antibacterial effects, inflammation modulation, or promotion of skin regeneration), which ultimately constrains their efficacy in tissue repair. (Wang et al., 2025) In response to these limitations, novel wound dressings equipped with stimuli-responsive capabilities and integrated multifunctionality have attracted significant research interest. (Niu et al., 2026) For example, electroactive dressings are easily prepared by integrating electroactive materials. (Wan et al., 2022) Unlike traditional dressings that merely provide passive isolation, electroactive dressings enable active regulation and actively promote wound healing.

Biological electricity is a fundamental regulator of cellular behavior and tissue function. (Hu et al., 2024) By mimicking endogenous bioelectric signals, the application of exogenous electrical stimulation (ES) has been employed in nerve, cardiac, and wound repair. (Sun et al., 2025) Endogenous electric field (EnEF) can accelerate wound-healing processes by promoting cell migration, proliferation, polarization, and angiogenesis. (Cui et al., 2026; Qin et al., 2025) Clinical research has validated that exogenous electric fields (ExEF) can similarly promote repair, positioning electroactive functional dressings as a uniquely promising therapeutic approach. By integrating electrical feature, these electroactive dressings replicate the native electrophysiological microenvironment, enabling them to actively participate in wound healing through anti-inflammatory, antibacterial, and angiogenic-promoting activities. (Du et al., 2024; Xin et al., 2026) Furthermore, their electro-responsive capability allows for on-demand, electrically triggered drug release. When combined with integrated biosensors, this technology paves the way for intelligent, closed-loop wound management.

**Scheme 1:**
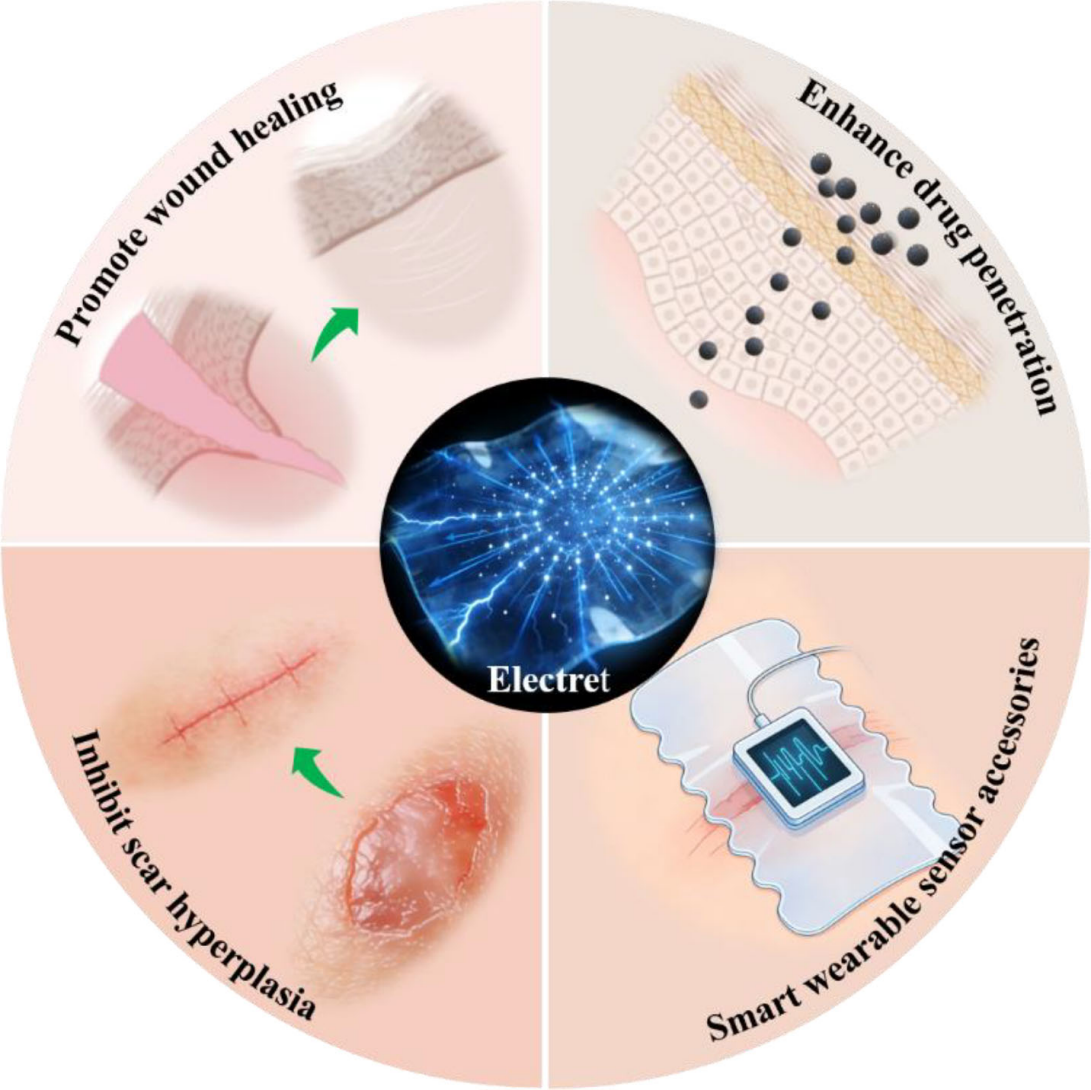
Application of electret-based dressings in wound treatment.

Traditional ExEF relies on external equipment for a continuous power supply, limiting its practicality for sustained therapeutic use. (Kaveti et al., 2026) Piezoelectric materials offer an alternative by generating charges in response to mechanical stress (Mao et al., 2026); however, within the physiological environment, the abundant free ions rapidly neutralize these piezoelectric charges, hindering the delivery of continuous electrical signals. Therefore, the key issue is how to ensure a stable power supply for the active dressing without an external source. In contrast, electrets can maintain a stable charge state after being poled by an external EF, thereby providing sustained ES even after the field is removed. (Li et al., 2024) An effective electret should exhibit semi-permanent or even permanent charge retention and remain stable against environmental factors. Beyond charge density and charge storage lifetime, the composition of the dielectric material itself is a critical determinant of electret performance. Currently, common organic polymer materials exhibit favorable electret properties, including polyethylene terephthalate, polyethylene, polypropylene, polytetrafluoroethylene, polyimide, fluorinated ethylene-propylene copolymer, and chitosan. These polymers are either highly insulating non-polar materials (ensuring superior charge storage) or strong polar materials with significant dipole moments. They possess varying degrees of biological activity or inertness and have been widely utilized in the repair and replacement of both hard and soft tissues. (Zhang et al., 2023b) Compared with inorganic counterparts, organic electrets offer superior flexibility and processability, enabling fabrication into ultra-thin, body-conforming structures that improve patient comfort and fluid management. More importantly, organic materials can be readily integrated with antibacterial, anti-inflammatory, and other biological functions through molecular design or physical blending strategies. This allows for the construction of a “smart active platform” that not only physically isolates external pathogens but also actively promotes cell proliferation. In terms of biocompatibility, organic electrets also offer superior advantages, thus better meeting the dual demands of comfort and efficient healing in modern wound care. This minireview includes the research progress on organic electrets and their application (**Scheme 1**) and regulatory mechanisms in wound healing. We hope this minireview will serve as a valuable reference for future research and clinical translation.

## 2 Recent advances in organic electrets for wound healing

### 2.1 Electret-based dressing for promoting wound healing

**Figure 1:**
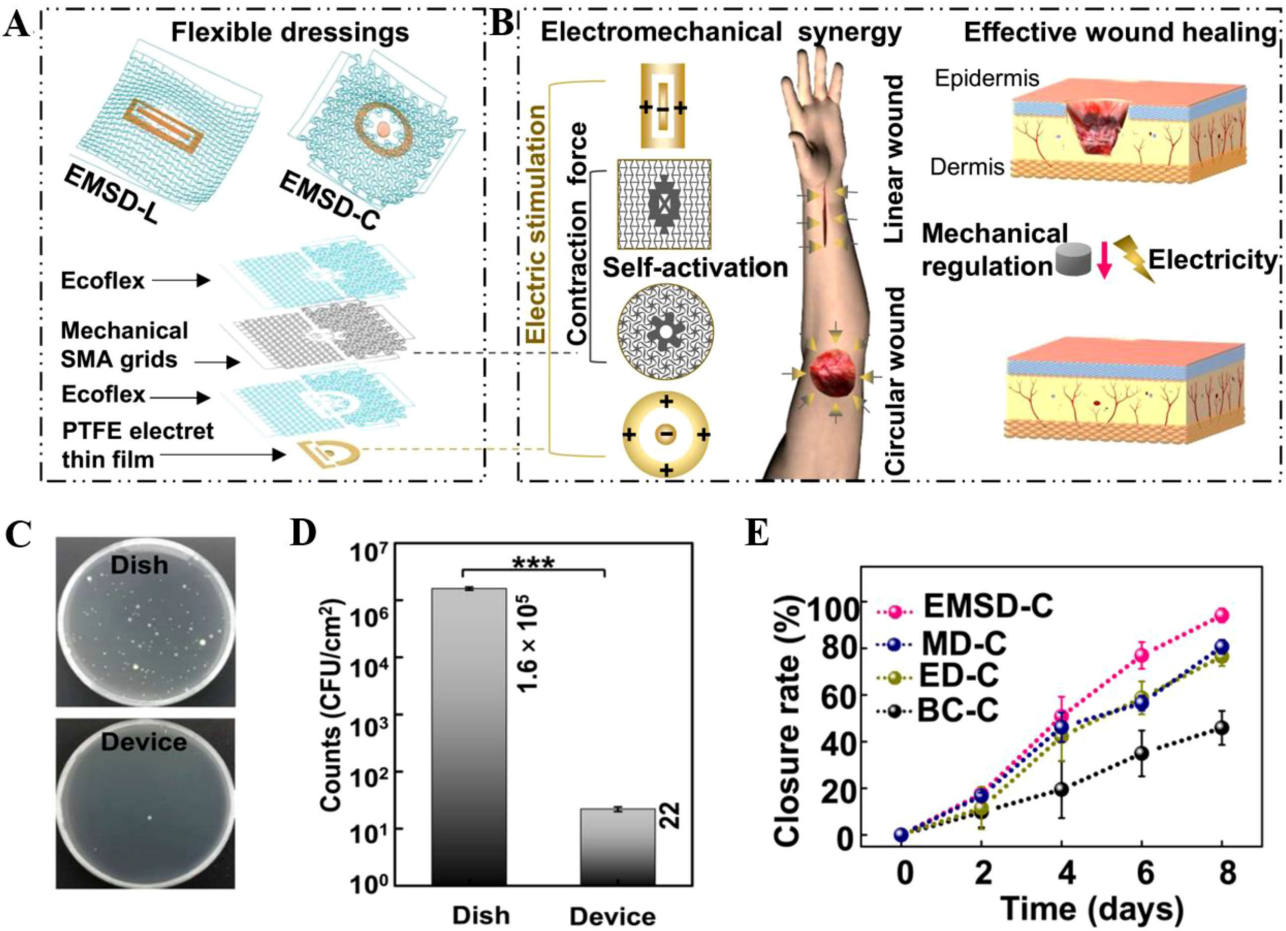
**(A)** Anatomical diagram of L(linear)/C(circular) EMSD bioelectronic dressing. **(B)** The operating principle of EMSDs. **(C)** Antibacterial coating plate and **(D)** colony count analysis of polarized (devise) and unpolarized (dish) PTFE film. **(E)** Statistics on wound closure. EMSD-C, MD-C, ED-C, and BC-C are circular electromechanical synergistic, EEF-based electrical, SMA-based mechanical, and blank control dressings, respectively. Reproduced with permission. (Kaveti et al., 2026) Copyright 2022, American Association for the Advancement of Science.

By mimicking the endogenous bioelectric field at wound sites, ES effectively guides cell migration, promotes angiogenesis, and regulates inflammatory responses, thus markedly accelerating tissue repair and regeneration. For example, Yao et al. synthesized a programmable, temperature-activated electromechanical collaborative wound dressing (EMSD), which consists of a mechanical metamaterial grid based on shape memory alloy (SMA) and a polarized polytetrafluoroethylene (PTFE) antibacterial electret electrostatic film (EEF). (**Figures 1A, B**) (Yao et al., 2022) Among these, the electret film is endowed with stable charges via electrospinning and polarization, generating a sustained external electrostatic field. This field not only enhances the endogenous transepithelial potential in injured tissue to direct directional migration of epithelial cells and promote re-epithelialization but also provides the dressing with a 99.99% bactericidal efficiency against *Escherichia coli* (**Figures 1C, D**). Meanwhile, the SMA grid undergoes a phase transition triggered by skin temperature (~35 °C), generating approximately 10% contraction strain. This exerts mild mechanical traction on the wound and promotes the secretion of vascular endothelial growth factor (VEGF). By using the electro-mechanical synergic action, the expression of VEGF, epidermal growth factor (EGF), and transforming growth factor-β (TGF-β) were upregulated, coordinately accelerating wound repair by modulating metabolic processes, promoting angiogenesis, and enhancing re-epithelialization. Mouse experiments confirmed rapid wound closure within 4 days for linear wounds and 8 days for circular wounds. The closure rates were over 50% higher than those of the blank control group and outperformed most existing non-physical intervention methods (**Figure 1E**).

### 2.2 Electret-based dressing for inhibiting scar formation

**Figure 2:**
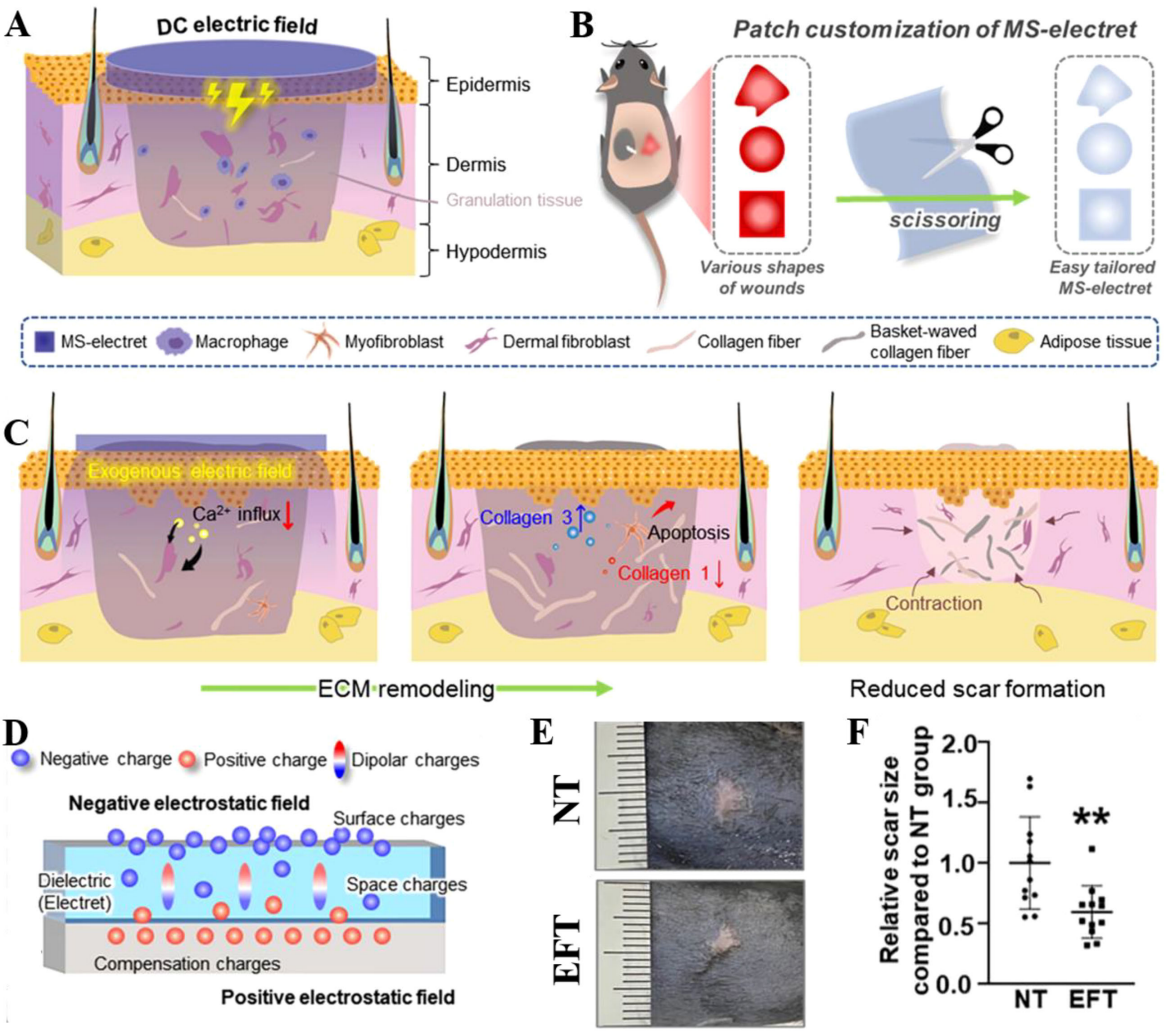
**(A)** MS-electret patch for scar inhibition by using a direct current (DC) electric field (EF). **(B)** The producibility of MS patch in different shapes. **(C)** The process of inhibiting scar formation of MS patch. **(D)** Polarization diagram of the FEP electret. Photographic records of scar formation **(E)** and the corresponding statistical data **(F)**. NT and EFT are no and EF treated groups. Reproduced with permission. (Kim et al., 2024) Copyright 2023, Wiley.

Statistics show a high incidence of pathological scarring during wound healing. (Kang et al., 2026) Hypertrophic scars develop in 30%–90% of burn patients, (Finnerty et al., 2016) while the incidence in ordinary wounds ranges from approximately 5% to 15% .(Jeschke et al., 2023) Future wound treatment should simultaneously promote healing and prevent scar formation. Previous studies have demonstrated that direct current EFs can effectively inhibit scar hyperplasia. Kim et al. fabricated multilayer stacked electrets (MS-electrets) from fluorinated ethylene-propylene (FEP) films via corona charging. (**Figures 2A, B**) (Kim et al., 2024) This structure yields a surface potential up to 3400 V and a stable direct-current EF lasting more than 5 days. Owing to its multilayer stacked architecture, the electric field intensity can be flexibly tuned and precisely tailored to match the wound contour. When applied for wound treatment, polarized FEP-based patch suppresses Ca²^+^ influx in human dermal fibroblasts and downregulates the activated T-cell nuclear factor pathway and JNK signaling pathways, and markedly reduces the expression of lysyl oxidase, type I collagen, and α-smooth muscle actin, thereby inhibiting fibroblast-to-myofibroblast differentiation (**Figures 2C, D**). This effectively prevents scar formation while promoting the structural regeneration of skin tissue (**Figures 2E, F**).

Beyond direct EF application, leveraging electret-generated fields to enhance the subcutaneous penetration of therapeutic agents represents another promising therapeutic strategy. (Secci et al., 2026) For example, Yuan et al. coated the anti-scarring drug 5-fluorouracil (5-FU) onto the surface of negatively charged polypropylene electrets to fabricate a drug-loaded patch. (Yuan et al., 2018) This patch leverages the electrostatic field and microcurrent generated by the electret to obviously enhance the transdermal penetration of 5-FU and its retention within scar tissue, thereby increasing the local drug concentration at the lesion site. Its mechanism for inhibiting hypertrophic scars involves enhanced 5-FU delivery, which reduces TGF-β1 expression, downregulates HSP47 levels, inhibits type I and III collagen synthesis, and decreases abnormal collagen deposition and fibroblast hyperproliferation, thus alleviating scar hypertrophy during wound healing.

**Figure 3:**
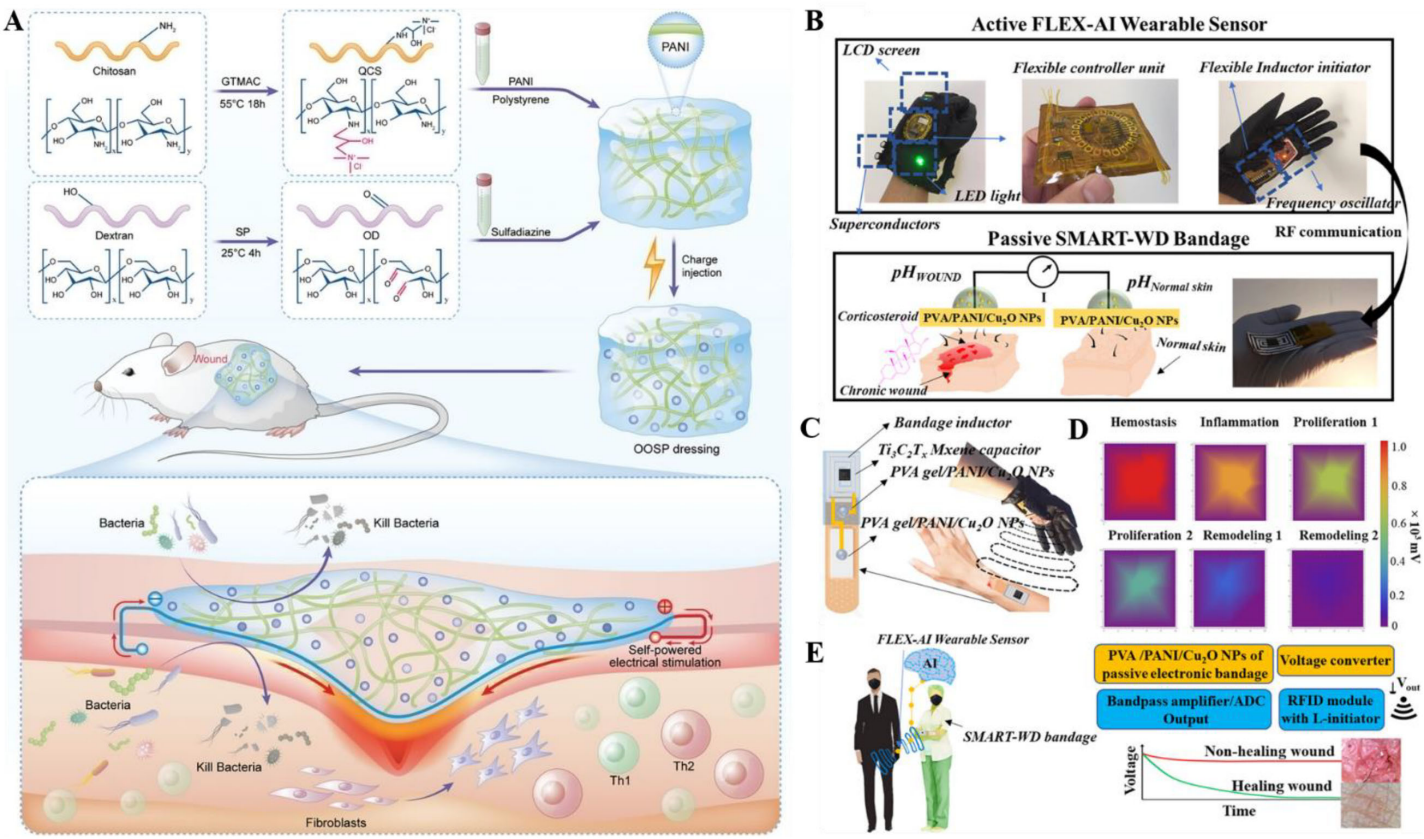
**(A)** Schematic illustration of the QOSP hydrogel and its mechanisms of action in electrotherapy and wound immune modulation. Reproduced with permission. (Liu et al., 2025a) Copyright 2025, Wiley. **(B)** The device framework of SMART-WD. **(C)** The anatomy map of electronic bandage. **(D)** Wound healing simulation by using the finite element method. **(E)** Schematic diagram of the SMART-WD application. Reproduced with permission. (Li et al., 2024) Copyright 2022, American Chemical Society.

However, most previous studies have focused exclusively on either promoting wound healing or suppressing scarring, without achieving the integration of both functions. To address this gap, Liu et al. synthesized the QOSP hydrogel from quaternized chitosan, oxidized dextran (OD), sulfonamide antibiotic (i.e., sulfadiazine), polystyrene, and polyaniline (PANI) nanowires, and implanted positive charges into the hydrogel through capacitively coupled plasma. (**Figure 3A**) (Liu et al., 2025a) The charge-injectable QOSP hydrogel patch with electret-like performances as polystyrene within the hydrogel acts as a charge-trapping medium, ensuring uniform distribution and long-term storage of the implanted charges. Then the biomimetic ES can be delivered to the wound via PANI conductive network, further enhancing cell proliferation, migration, and differentiation. Moreover, 90% of Gram-negative and Gram-positive bacteria can be eliminated by the combined action of sulfadiazine and quaternized chitosan, as quaternary ammonium cations bind to and disrupt negatively charged bacterial membranes. Meanwhile, the quaternized chitosan component in the hydrogel can downregulate inflammatory and pro-fibrotic pathways and drive the Th1/Th2 balance toward an anti-fibrotic Th1 phenotype, reducing excessive fibrosis. In addition, the QOSP hydrogel accelerates healing by promoting wound transition from inflammation to proliferation, thereby achieving the dual therapeutic effects of accelerated burn wound healing and scar inhibition.

### 2.3 Electret-based dressing for wound monitoring

Wound dressings with detection capabilities have made significant progress. These wearable dressings monitor changes in physical and chemical biomarkers (e.g., temperature, pH, uric acid, glucose) by using optical or electrical detection methods during wound healing. (Tang et al., 2021; Wu et al., 2023) Additionally, self-healing materials, wireless transmission, drug-controlled release systems, and artificial intelligence-assisted diagnosis can be introduced into the dressings to achieve multi-functional and personalized wound management. However, A persistent challenge in wound care is the real-time monitoring of healing status and the formulation of personalized treatment plans based on dynamic conditions. (Yuan et al., 2025; Zhao et al., 2025) Kalasin et al. further extended the function of electrets from treatment to monitoring. (Kalasin et al., 2022) They developed a binary wireless monitoring system composed of a wearable pH sensor and an intelligent wound dressing (SMART-WD) (**Figure 3B**). Laser-etched Mxene–polytetrafluoroethylene composite electret was used as a flexible capacitive element. This component is embedded in the bandage and establishes radiofrequency coupling with the sensor antenna, enabling battery-free operation and wireless signal transmission. The dressing features a pH-sensitive interface (sensor) fabricated by modifying PANI and copper oxide nanoparticles on copper electrodes, followed by coating with polyvinyl alcohol (PVA) hydrogel (**Figure 3C**). During the healing process, local pH variations induce hydrogen ion migration and trigger interfacial reactions at the PANI/Cu_2_O junction, which will generate electrical signals that are wirelessly transmitted via the electret antenna. By combining with a deep artificial neural network, the sensor can identify the inflammatory, proliferative, and remodeling phases with an accuracy of up to 94.6% (**Figure 4D**). At the same time, this wound dressing can promote collagen deposition, thereby achieving the dual goals of precise monitoring and accelerating tissue regeneration (**Figure 3E**).

## 3 Conclusion and perspective

During wound healing, the disruption of EnEFs represents a critical barrier to effective regeneration. The introduction of bioelectrically active functional materials as exogenous signal sources provides a promising strategy to modulate the local electrical microenvironment and enhance tissue repair. As long-term polarized functional dielectrics, electrets exert biological effects through stable electrostatic fields and sustained, non-invasive electrical signal output, enabling stable long−term regulation at the wound site. This minireview summarizes recent advances in electret-based dressings for wound healing. Despite substantial progress and encouraging application potential, the clinical translation of electret−based therapies still faces significant challenges.

1. Biocompatibility: Biocompatibility represents an indispensable prerequisite for the *in vivo* implementation of electret-based dressings. (Chai et al., 2025; Salvatore et al., 2026) Although the majority of electrets display satisfactory biocompatibility, further refinement of their surface characteristics is essential to mitigate adverse biological responses, including immune rejection and inflammatory activation. Moreover, conventional polymer−based electrets (e.g., polytetrafluoroethylene, FEP, polypropylene, and polystyrene) are typically non−biodegradable, giving rise to potential ecological and long−term biosafety concerns (Salvatore et al., 2026). For example, these non−biodegradable polymer remaining at wound sites for a long time may cause chronic inflammation, foreign body reactions, or cellular metabolic disorders. In practice, surgical dressing removal often causes secondary wound damage. Future research should focus on biodegradable electrets. Current biodegradable electrets (e.g., polylactic acid, chitosan) have inferior charge retention to pure organic electrets. Therefore, the development of degradable electrets derived from natural biomaterials—such as polysaccharides, amino acids, and proteins—while retaining robust charge−storage performance, represents a pivotal direction for the sustainable advancement of biodegradable electret−based dressing.
2. Enhancement of polarization stability and volumetric charge density: In practical applications, electret−based dressings must sustain stable polarization charges throughout the extended timeline of wound healing. In biodegradable systems, the balance between charge retention and degradation kinetics must be carefully regulated. In most existing electrets, charges remain confined to the surface with limited density and this limits therapeutic efficacy. The construction of bulk electrets with porous architectures or three-dimensional networks (e.g., electret-based hydrogels and multilayer electret films) offers a promising route to increase volumetric charge density and improve charge storage stability.
3. The tailoring of the EF to physiological spatiotemporal scales: Electrical signals generated by electrets must be precisely tuned in intensity and duration to match the spatiotemporal physiological characteristics of EnEFs. Therefore, introducing a charge management electronic module into electret-based dressings better simulates the natural bioelectrical environment, which is essential to maximize exogenous ES for effective tissue repair.
4. Elucidation of regenerative mechanisms: While ES is known to drive tissue regeneration by regulating membrane potential, ion channels, and core signaling pathways (e.g., calcium, Wnt, PI3K/Akt, and MAPK). (Hao et al., 2025; Xue et al., 2025b) However, the precise electret-mediated regulation mechanisms remain largely elusive. Therefore, future research should integrate materials science and electrophysiology to elucidate the interactions between electrets and biological systems, thereby enabling more precise spatiotemporal control over wound healing.
5. Simplification of charging techniques and scalability of fabrication: Charge polarization and injection represent fundamental processes underlying the functionality of electret materials. Multiple techniques have been established, including X−ray irradiation, corona discharge, thermal polarization, contact charging, and electron beam injection. (Liu et al., 2026) Apart from corona discharge, the majority of these approaches are limited by complicated operational procedures and restricted scalability. The development of simple, efficient, and industrially scalable polarization technologies is critical for the further commercial translation of electret-based dressings.

In summary, electret-based dressing promotes healing via an electrostatic field, which can reduce dressing change frequency and infection risk. Meanwhile, this dressing is expected to not only lower medical costs and shorten hospital stays but also drive growth in the high-end medical dressing market, offering significant clinical and economic value. Yet further advances are required in biocompatibility, charge stability, mechanistic understanding, application diversity, and fabrication scalability. Future work should promote interdisciplinary collaboration between materials science, electrophysiology, and clinical medicine to resolve these challenges in wound healing.
